# Variant Type X91^+^ Chronic Granulomatous Disease: Clinical and Molecular Characterization in a Chinese Cohort

**DOI:** 10.1007/s10875-022-01324-3

**Published:** 2022-07-07

**Authors:** Bijun Sun, Zeyu Zhu, Xiaoying Hui, Jinqiao Sun, Wenjie Wang, Wenjing Ying, Qinhua Zhou, Haili Yao, Jia Hou, Xiaochuan Wang

**Affiliations:** 1 Department of Clinical Immunology, Children’s Hospital of Fudan University, 399 Wanyuan Road, Shanghai, 201102 China; 2 Department of Clinical Medicine, Shanghai Medical College, Fudan University, Shanghai, 200032 China; 3 Department of Pediatrics, Shanghai Songjiang District Central Hospital, Shanghai, 201600 China; 4 Shanghai Institute of Infectious Disease and Biosecurity, Shanghai, 200032 China

**Keywords:** Chronic granulomatous disease, gp91phox, Neutrophil respiratory burst, Stimulation index, NADPH oxidase

## Abstract

**Purpose:**

We aimed to report the clinical and immunological characteristics of variant type X91^+^ chronic granulomatous disease (CGD) in a Chinese cohort.

**Methods:**

The clinical manifestations and immunological phenotypes of patients with X91^+^ CGD were collected. A dihydrorhodamine (DHR) analysis was performed to evaluate neutrophil function. Gp91^phox^ protein expression was determined using extracellular staining with the monoclonal antibody (mAb) 7D5 and flow cytometry.

**Results:**

Patients with X91^+^ CGD accounted for 8% (7/85) of all patients with CGD. The median age of onset in the seven patients with X91^+^ CGD was 4 months. Six patients received the BCG vaccine, and 50% (3/6) had probable BCG infections. *Mycobacterium tuberculosis* infection was prominent. The most common sites of infection were the lung (6/7), lymph nodes (5/7), and soft tissue (3/7). Two patients experienced recurrent oral ulcers. The stimulation index (SI) of the patients with X91^+^ CGD ranged widely from 1.9 to 67.3. The difference in the SI among the three groups of patients (X91^+^ CGD, X91^−^ CGD, and X91^0^ CGD) was statistically significant (*P* = 0.0071). The three groups showed no significant differences in onset age, diagnosis age, or severe infection frequency. *CYBB* mutations associated with X91^+^ CGD were commonly located in the second transmembrane or intracellular regions. Three novel X91^+^ CGD–related mutations (c.1462–2 A > T, c.1243C > T, and c.925G > A) were identified.

**Conclusions:**

Variant type X91^+^ CGD may result in varied clinical manifestations. Moreover, the laboratory findings might indicate a moderate neutrophil SI. We should deepen our understanding of variant X91^+^ CGD to prevent missed diagnoses.

**Supplementary Information:**

The online version contains supplementary material available at 10.1007/s10875-022-01324-3.

## Introduction

Chronic granulomatous disease (CGD) is a rare inherited primary immunodeficiency (PID) with an incidence of 1 case per 200,000 to 250,000 live births worldwide [1]. The pathogenesis of CGD is characterized by dysfunctional nicotinamide adenine dinucleotide phosphate (NADPH) oxidase and decreased or no superoxide anion (O_2_^−^) production, resulting in significantly reduced bactericidal activities of neutrophils and other phagocytes. The clinical patterns of patients with CGD generally include early-onset recurrent bacterial, tuberculous, or fungal infection, especially with catalase-positive microbes, involving the lung, lymph nodes, liver, and bone [2]. The respiratory burst of neutrophils has been previously detected using the nitroblue tetrazolium (NBT) test and is currently detected with the dihydrorhodamine-1,2,3 (DHR) test in clinical practice. The respiratory burst of neutrophils is defective in patients with CGD.

NADPH oxidase is a complex composed of membrane-bound gp91phox and p22phox and cytosolic p47phox, p67phox, and p40phox. Gp91phox and p22phox together form the heterodimer cytochrome b558. Gp91phox is the enzymatic core of the NADPH oxidase complex, while other components play a regulatory role in the NADPH oxidase complex. The gp91phox protein is encoded by the cytochrome b-245 β chain (*CYBB*) gene mapped to chromosome Xp21.1-p11.4, and its mutation results in X-linked CGD, accounting for 70% of CGD cases overseas and approximately 85% of cases in China [1, 3]. Most patients with X-linked CGD neither produce superoxide nor express gp91phox, and this type of CGD is named classic type X-linked CGD (X91^0^ CGD). However, some patients with *CYBB* mutations have low expression levels of functional or partially functional gp91phox (X91^**−**^ CGD) or normal expression levels of nonfunctional or partially functional gp91phox (X91^**+**^ CGD) [1, 4]. X91^−^ CGD and X91^+^ CGD caused by *CYBB* mutations are named variant type X-linked CGD. The characteristics of variant type X-linked CGD, especially X91^+^ CGD, differ from classical CGD, which may interfere with the rapid diagnosis of X-linked CGD using the stimulation index (SI) of DHR and the expression of the gp91 protein.

However, few patients with X91^+^ CGD have been reported to date. In this study, we report the clinical and immunological characteristics of seven Chinese patients with X91^+^ CGD, focus on the relationship with SI, and review the literature to further document the rare *CYBB* gene mutations resulting in variant type X-linked CGD.

## Methods

This study was approved by the Ethics Committee of Children’s Hospital of Fudan University. Written informed consent was obtained from the parents of all patients.

### Patients and Clinical Data

Children with CGD who were admitted to our hospital between January 2018 and March 2021 were enrolled in this study. CGD was diagnosed according to the clinical manifestations, laboratory findings, and genetic data. The relevant data are summarized in detail in Table 1. Previous studies of patients with variant type X91^+^ CGD and mutations reported in PubMed from 1987 to November 2021 were reviewed.

**Table 1:** Baseline characteristics of patients with X91^+^ CGD

Characters	Patient 1	Patient 2	Patient 3	Patient 4	Patient 5	Patient 6	Patient 7
Gender	M	M	M	M	M	M	M
Age of onset	3 m	12 m	0.7 m	4 m	5 m	0.5 m	24 m
Age of diagnosis	11 m	84 m	4 m	22 m	108 m	2 m	60 m
Age of last follow-up	13 m	138 m	12 m	31 m	130 m	15 m	105 m
Clinical presentation	BCG disease, axillary lymph node enlargement, recurrent pneumonia, diarrhea, soft tissue infection, osteomyelitis, sepsis, impaired liver function	Recurrent aphthous ulcer, soft tissue infection, cervical lymphadenitis, pneumonia	Recurrent pneumonia, rashes, impaired liver function	BCG disease, Recurrent fever, axillary lymph node enlargement, recurrent pneumonia, perianal abscess	BCG disease, lymph node enlargement, upper respiratory infection, otitis media	Recurrent fever, recurrent pneumonia, pulmonary abscess, perianal abscess, recurrent diarrhea, sepsis, skin and soft tissue infection	Recurrent fever, lymph node enlargement, pneumonia, oral ulcer
Pathogenic microorganism	BALF*: GM* + *;* Pus: *Burkholderia gladioli (reads, 69);* Blood: *Mycobacterium neoaurum (reads, 78);* Stool: *Clostridium difficile*	Negative	Negative	Sputum: *Ureaplasma urealyticum*; BALF: *Corynebacterium striatum (reads, 200)*	Negative	Skin pus: *Klebsiella pneumoniae*	Negative
Treatment	Meropenem, vancomycin, voriconazole, SMZ, etc.; IVIG	Azithromycin, cefoperazone/sulbactam, rifampin, clarithromycin, SMZ, voriconazole; IFN-γ	Cefoperazone/sulbactam, moxalactam, imipenem, vancomycin, fluconazole, itraconazole and SMZ; IVIG	Isoniazide, rifampicin with ethambutol,linezolid, cefoperazone/sulbactam, azithromycin, itraconazole, SMZ	Isoniazide, rifampicin, ethambutol, pyrazinamide	Ceftriaxone, meropenem, cefoperazone/ sulbactam, fluconazole, vancomycin, cefaclor, SMZ, isoniazide, rifampicin, itraconazole; IVIG	Isoniazide, rifampicin, ethambutol, pyrazinamide, linezolid, itraconazole; IFN-γ
Outcome	HSCT	Anti-infection	HSCT	Anti-infection	Anti-infection	HSCT	Anti-infection
SI	67.3	35.4	21.2	1.9	40.7	17.1	61.2
GP91 protein	X91 +	X91 +	X91 +	X91 +	X91 +	X91 +	X91 +
CYBB Gene Mutation	c.162G > C	c.1462–2 A > T	c.170C > A	c.1223G > A	c.1243C > T	c.1244C > A	c.925G > A
Amino acid change	p.R54S	partial del exon 12 p.Ala488_Glu497del	p.A57E	p.G408E	p.P415S	p.P415H	p.E309K
SIFT score	0.001		0	0.001	0	0	0.002
Polyphen2 score	0.904		0.999	1	1	1	0.998
MutationTaster score	1	1	1	1	1	1	1
MAF in Gnomad	0	0	0	0	0	0	0

*M*, male; *m*, months; *BCG*, Bacillus Calmette-Guerin; *GM*, galactomannan detection; *BALF*, bronchoalveolar lavage fluid; *SMZ*, sulfamethoxazole; *IVIG*, intravenous immunogloblin; *IFN-γ*, interferon-γ; *HSCT*, hematopoietic stem cell transplantation; *MAF*, minimum allele frequency

#### Definition of Terms

The age of the last follow-up was defined as the pretransplant visit for transplant patients, and November 2021 or death for nontransplant patients. Severe infection events were defined as infections requiring hospitalization for systemic anti-infective therapy or surgical treatment. The frequency of severe infection events per patient-year refers to the total number of severe infection events/the duration of the effective observation years. Sites of infections were described according to the clinical manifestations: lung, lymph node, perianal, gastrointestinal tract, bone, bloodstream, skin and soft tissue, liver, spleen, abdominal (abdominal abscess and peritonitis), central nervous system, and urinary tract. Noninfectious complications included oral ulcers, elevated levels of liver enzymes, ulcerative colitis, and anemia. The diagnostic criterion for BCG disease was described in a previous publication [2]. *Mycobacterium tuberculosis* infection was considered based on pulmonary and lymph node infection characteristics, a positive PPD test, T-SPOT assay, and effectiveness of anti-tuberculosis treatment. Here, we defined the calculation of *M. tuberculosis* infection as the total number of patients receiving anti-tuberculosis treatment and those with BCG disease. PPD positivity was defined as an induration diameter > 5 mm after 48–72 h. Fungal infection was defined as a positive galactomannan (GM) test or the isolation of a fungus from samples. Bacterial infection was defined as detecting pathogenic bacteria using cultures or metagenomics next-generation sequencing (mNGS).

### Routine Immune Function Evaluation

Routine blood counts and immunological functional analyses were performed. We used the nephelometry method to detect immunoglobulins, including IgG, IgA, and IgM, as previously reported, and lymphocyte subsets were measured using flow cytometry (Becton Dickinson, Franklin Lakes, NJ, USA).

### Evaluation of Neutrophil Function

A DHR analysis was performed to measure the respiratory burst of neutrophils by assessing reactive oxygen species (ROS). Blood was collected and treated with the anticoagulant heparin sodium. Samples were stimulated with 0.01 mg/ml phorbol-12-myristate-14-acetate (PMA) for 15 min and then incubated with dihydrorhodamine-1,2,3 (0.03 mg/ml) for 5 min, followed by red blood cell lysis and PBS washes [2]. The flow cytometry analysis was performed as previously described using a FACSCanto II flow cytometer (Becton Dickinson, Franklin Lakes, NJ, USA) [5]. Since we purchased a new flow cytometer in January 2021, the channel template set was AF488 from January 2008 to December 2020, and the channel template set changed to FITC from January 2021. The stimulation index (SI), which is defined as the geometric fluorescence intensity of PMA-stimulated neutrophils relative to the geometric fluorescence intensity of unstimulated neutrophils, was calculated to evaluate neutrophil function [6]. The SI was detected by monitoring the FITC channel in only one patient with X-linked CGD (P4).

### Expression of the gp91^phox^ Protein

The expression of the gp91^phox^ protein was determined using extracellular staining with the monoclonal antibody (mAb) 7D5, which detects flavocytochrome b558 in the NADPH oxidase complex at the cell surface, and flow cytometry. Peripheral blood samples were added to two tubes. FITC anti-flavocytochrome b558 (MBL) or an isotype-matched control antibody (BD Biosciences) was added to the reaction system and incubated for 15 min for staining, followed by red blood cell lysis and PBS washes. The expression of gp91phox protein on peripheral blood neutrophils was analyzed using a FACSCanto II flow cytometer. Each time we performed the experiment, we included a sample from a healthy control in the same batch. The gp91phox protein was detected using the FITC channel in three patients, including one with X91^0^ and two with X91^+^ (P1 and P4).

### Gene Analysis

Genomic DNA was extracted from the EDTA-anticoagulated blood of patients and their parents using the QIAmp® DNA Blood Mini Kit (Qiagen, Hilden, Germany). The concentration and quantity of the DNA samples were measured using a NanoDrop ultraviolet spectrophotometer (Thermo Fisher Scientific, Waltham, MA, USA) and then prepared for next-generation sequencing by a panel including all previously reported immunodeficiency genes.

In accordance with the instructions of the SureSelect Human All Exon Kit, the genomic DNA underwent ultrasonic fragmentation, end repair, adapter connection, and hybridization. The captured DNA library was sequenced on the Illumina HiSeq 2000 platform (Illumina, San Diego, CA). The raw data were converted to a VCF file containing the basic information for the mutation sites through splicing and comparison. We completed the variation annotation using ANNOVAR and VEP software. The annotation of the mutation frequency referred to databases, including the 1000 Genomes Project, the ExAC Browser, and internal databases. Mutations were predicted using SIFT, PolyPhen-2, and MutationTaster software. Deletions were identified by performing an NGS coverage depth analysis [7], and point mutations were confirmed by Sanger sequencing. The minimum allele frequency (MAF) was searched in the Gnomad database.

### Statistical Analysis

Data were analyzed using STATA 15.0 software. Categorical variables were displayed as numbers and percentiles. We used the Kruskal–Wallis rank sum test to analyze the differences among the three groups (X91^+^, X91^−^, and X91^0^ CGD). The specific source of the significant differences was then determined by a two-by-two comparison between the two groups. The tests were Bonferroni corrected. Pearson correlation analysis was used to assess the correlation between the two quantitative variables. *P* values < 0.05 were considered statistically significant.

## Results

### X-linked CGD Distribution

**Fig. 1:**
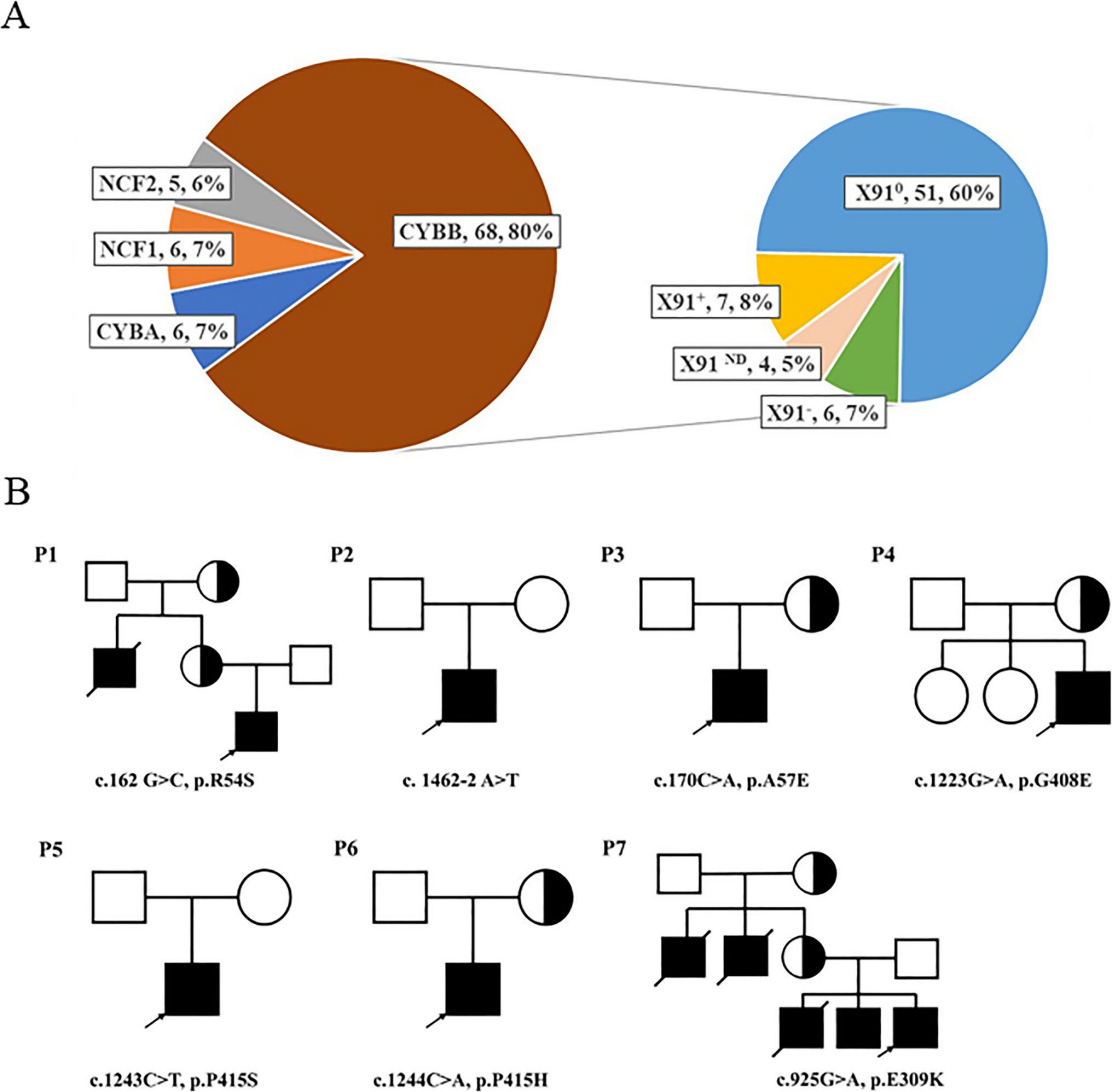
Proportion of patients with X91^+^ CGD and family tree. **A** Distribution of genes and the GP91 protein in patients with CGD. **B** The pedigrees of the 7 Chinese patients with X91^+^ CGD

Between January 2018 and March 2021, a total of 85 patients with CGD were admitted to the Children’s Hospital of Fudan University. Patients were diagnosed with CGD according to their clinical manifestations, immune phenotype, and genetic results. The most common mutation in the *CYBB* gene inherited from the X chromosome was detected in 68 patients (80%). Autosomal recessive CGD (AR-CGD) was detected in a minority of the patients and was attributed to *CYBA* mutations (7%), *NCF1* mutations (7%), and *NCF2* mutations (6%) (Fig. 1A).

**Fig. 2:**
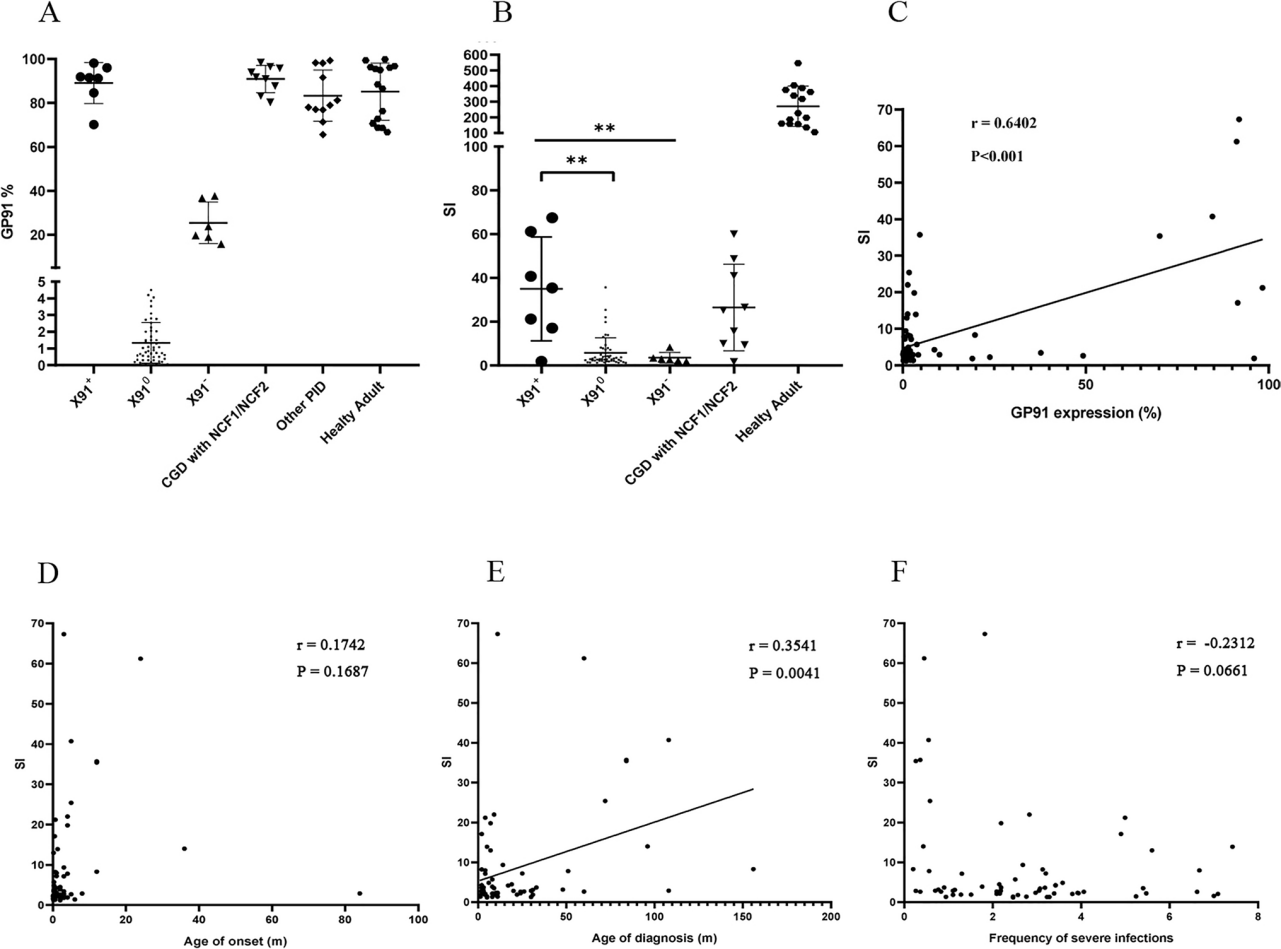
Comparison of GP91 protein expression and stimulation index in patients with X-linked CGD. **A** GP91 protein expression levels in different groups, including X91^+^, X91^−^, X91^0^, CGD with NCF1/NCF2, other PIDs, and healthy adults. **B** SI in different groups of patients with X-linked CGD and healthy adults. **A statistically significant difference was observed among the three groups (X91^+^, X91^−^, and X91^0^ CGD) (*P* = 0.0071). Further analysis showed that the SI of X91^+^ CGD was statistically higher than that of X91^0^ CGD (*P* = 0.002). **C** Correlation analysis between SI and GP91 protein expression (*P* < 0.001). **D** Correlation analysis between SI and age of onset (*P* = 0.1687). **E** Correlation analysis between SI and age of diagnosis (*P* = 0.0041). **F** Correlation analysis between SI and frequency of severe infections (*P* = 0.0661). GP91 protein levels in three patients and the SI in one patient were detected using the FITC channel after changing to a new flow cytometer, including one patient with X91^0^ CGD and two patients with X91^+^ CGD (SI of 1.9 for P4)

Based on the expression level of the gp91 protein, we further classified patients with X-linked CGD carrying *CYBB* mutations into X91^0^, X91^−^, and X91^+^ CGD. In comparisons to both healthy controls and other primary immunodeficiencies (PIDs) including NCF1/NCF2 mutant CGD, we defined the gp91 expression level in patients with X91^+^ as > 70%, X91^−^ as 5–70%, and X91^0^ as < 5%. X91^+^ CGD accounted for 8% (7/85) of all CGD patients (Fig. 2A).

### Clinical Manifestations of X91^+^ CGD

#### Overview

Here, we report seven patients with X91^+^ CGD exhibiting different neutrophil stimulation indices and varying clinical presentations (Table 1). The median age of onset was 4 months (range, 0.5–24 months), and two patients had early clinical manifestations during the neonatal period. The median age of diagnosis was 22 months, and three patients were diagnosed after 5 years of age. None of the patients were products of a consanguineous union and two (P1 and P7) had a positive family history (Fig. 1B). The uncle of patient 1 (P1) died of unknown causes at the age of 10 years. Two uncles and one oldest brother of P7 died of fever and pulmonary infection within the age of 5 years. The second brother of P7 presented with regional BCG infection and recurrent pulmonary tuberculosis.

#### Infection Characteristics

Infections were the most common clinical symptoms and varied in severity. The patients had different sites of infections, such as lung (6/7), lymph nodes (5/7), skin and soft tissue (3/7), perianal (2/7), blood stream (2/7), bone (1/7), and gastrointestinal tract (1/7) infections. Pneumonia was common, most with recurrent infections. In terms of the etiology, these patients were at high risk for *M. tuberculosis* (5/7), bacterial (3/7), and fungal (1/7) infections (supplemental data 1: S1). One patient was considered to have a definite fungal infection due to a positive galactomannan (GM) test in bronchoalveolar lavage fluid (BALF). Bacteria including *Burkholderia gladioli*, *Corynebacterium striatum*, and *Klebsiella pneumoniae* were detected in the pus or respiratory secretions by culture or mNGS. *Mycobacterium neogold* (reads, 78) was detected in the blood of one patient with osteomyelitis and multiple bone destruction.

*M. tuberculosis* infection was considered a prominent manifestation of X91^+^ CGD patients. With the exception of P3, all patients received the BCG vaccine at birth, according to the vaccination records, and 50% (3/6) had probable BCG infections. P1 and P4 presented with left axillary lymph node enlargement at 3–4 months old with delayed healing of BCG vaccination sites. P4 had persistent lymph node enlargement with intermittent fever, and his chest CT showed bilateral lung infection. His PPD test was strongly positive (+ + +) at the age of 15 months. P5 presented with suppuration at the BCG vaccination site at 5 months, followed by clavicular and scapular lymph node enlargement. At the age of 7, he developed recurrent fever with cervical lymph node enlargement. The pathology of the lymph node biopsy was suggestive of tuberculosis, and the PPD test was positive. P6 had a chest CT showing diffuse bilateral lung lesions and a positive PPD test (+ +); therefore, *M. tuberculosis* was considered. P7 suffered from recurrent fever and lymph node enlargement beginning at the age of 4 years, and his chest CT was notable for miliary tuberculosis and lymph node calcification. He also had a positive PPD test.

#### Noninfectious Complications

Two patients (P2 and P7) experienced recurrent oral ulcers. Two had a temporary liver function impairment characterized by elevated liver enzyme levels. Lastly, two patients (P1 and P6) developed anemia.

#### Treatment and Follow-up

Most patients (5/7) received prophylactic sulfamethoxazole (SMZ). Five symptomatic patients received double, triple, or quadruple anti-tuberculosis medications consisting of isoniazide and rifampicin with or without ethambutol and pyrazinamide, and responded to the treatment. Six patients underwent antifungal treatment, including voriconazole, fluconazole, or itraconazole. Two patients (P2 and P7) received interferon gamma (IFN-γ) therapy and were in good condition during the follow-up period. Three patients received hematopoietic stem cell transplantation (HSCT), and all survived. Four patients who did not receive HSCT were still alive, as confirmed by clinical follow-up.

### Comparison Between X91^+^ and Other X-linked CGD Types

**Fig. 3:**
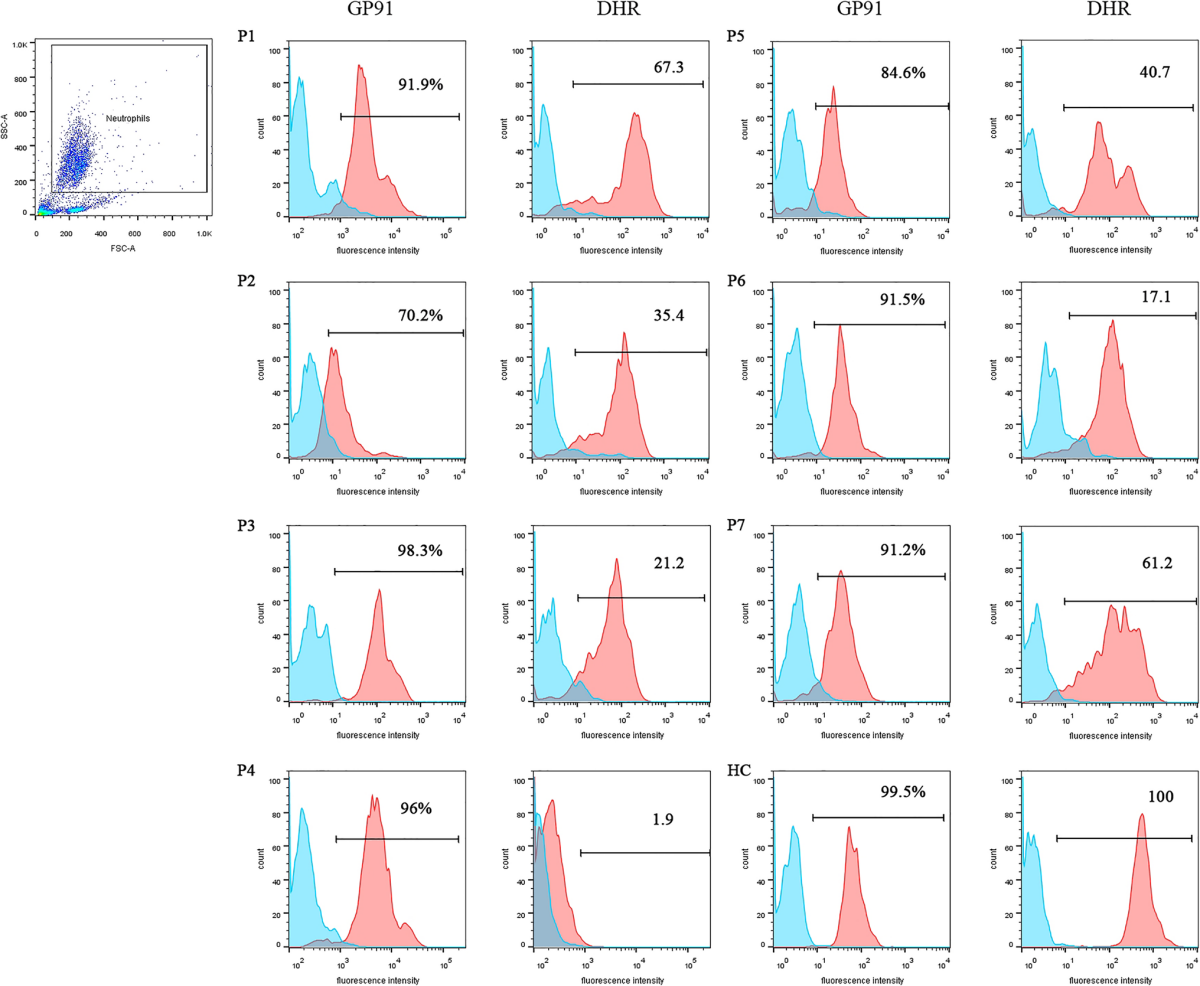
GP91 expression and the DHR assay for patients 1–7 with X91^+^ CGD. Gp91 assay: isotype control (blue) and GP91 antibody (red) are shown. The *Y*-axis represents the cell count. The *X*-axis is the fluorescence intensity. The % represents the expression of gp91 on the patient compared to the control. DHR assay: unstimulated (blue) and PMA-stimulated (red) neutrophils are shown. The *Y*-axis represents the cell count. The *X*-axis is the fluorescence intensity. The SI of each patient is marked. HC: healthy control. Samples from P1 (gp91 expression) and P4 (gp91 expression and DHR) were analyzed with the FITC channel, and the other samples were analyzed using the AF488 channel

Patients with X-linked CGD carrying *CYBB* mutations were divided into classic type X-linked CGD (X91^0^ CGD) and variant type X-linked CGD, including X91^−^ CGD and X91^+^ CGD based on gp91 expression (Fig. 2A). In our cohort, the SI of the seven patients with X91^+^ CGD varied widely from 1.9 to 67.3, including five with an SI ≥ 20 (Fig. 2B and Fig. 3). The SI ranged from 1.2 to 35.7 in patients with X91^0^ CGD, including 74.5% (38/51) of patients with an SI ≤ 5, while the SI in patients with X91^−^ CGD was less than 5, with only one exception. The difference in the SI among the three groups (X91^+^ CGD, X91^−^ CGD, and X91^0^ CGD) was statistically significant (*P* = 0.0071), and a higher SI was associated with higher gp91 protein expression (*r* = 0.6402, *P* < 0.001) (Fig. 2C). Correlation analysis between the SI and the age of onset (*P* = 0.1687), the age of diagnosis (*P* = 0.0041), and the frequency of severe infections (*P* = 0.0661) was performed (Fig. 2D, E, and F).

We further compared the clinical characteristics of the three groups (X91^+^ CGD, X91^−^ CGD, and X91^0^ CGD) in terms of onset age, diagnosis age, infection frequency, infection sites, and other parameters (Table 2). Patients with X91^0^, X91^−^, and X91^+^ CGD showed no significant differences in onset age, diagnosis age, or severe infection frequency. Lung, lymph node, and skin infections were common infection sites in patients with X91^+^, X91^0^, and X91^−^ CGD. Approximately half of the patients with X91^+^ and X91^0^ CGD had definite or probable BCG infections. Among patients without HSCT treatment, all patients with X91^+^ and X91^−^ CGD survived, but two patients with X91^0^ CGD died.

**Table 2:** Clinical characteristics of patients with X91^+^, X91^−^, and X91^0^ CGD

		X91 ^+^	X91 ^−^	X91 ^0^	*P* value
Patients, n		7	6	51	
Gender, male		7	6	51	
Age of onset, median (min, max)		4 m (0.5–24)	2 m (0.4–84)	1 m (0.1–36)	0.1092
Age of diagnosis, median (min, max)		22 m (2–108)	10 m (2–156)	9 m (1–96)	0.4029
Age of last follow-up, median (min, max)		31 m (12–138)	50 m (11–176)	28 m (2–164)	0.4899
Frequency of severe infections*, (mean ± SD)		2.07 ± 2.05	2.86 ± 2.68	2.78 ± 1.81	0.4457
Sites of infections					
	Lung	85.7% (6/7)	100% (6/6)	100% (51/51)	
	Lymph node	71.4% (5/7)	50% (3/6)	56.9% (29/51)	
	Skin and soft tissue	42.9% (3/7)	33.3% (2/6)	49% (25/51)	
	Gastrointestinal tract	14.3% (1/7)	50% (3/6)	21.6% (11/51)	
	Perianal	28.6% (2/7)	50% (3/6)	41.2% (21/51)	
	Blood stream	28.6% (2/7)	33.3% (2/6)	49% (25/51)	
	Abdominal	14.3% (1/7)	33.3% (2/6)	5.9% (3/51)	
	Bone	14.3% (1/7)	16.7% (1/6)	15.7% (8/51)	
	Central nerve system	0% (0/7)	16.7% (1/6)	5.9% (3/51)	
Pathogen of infection					
	Bacteria	42.9% (3/7)	83.3% (5/6)	66.7% (34/51)	
	Fungus	14.3% (1/7)	0% (0/6)	58.8% (30/51)	
	Mycobacterium tuberculosis	71.4% (5/7)	33.3% (2/6)	68.6% (35/51)	
	BCG infection	42.9% (3/7)	33.3% (2/6)	54.9% (28/51)	
	Mycoplasma	14.3% (1/7)	16.7% (1/6)	13.7% (7/51)	
Non-infectious complications					
	ulcer colitis	0% (0/7)	16.7% (1/6)	5.9% (3/51)	
	oral ulcers	28.6% (2/7)	33.3% (2/6)	11.8% (6/51)	
Treatment					
	SMZ	71.4% (5/7)	100% (6/6)	100% (51/51)	
	Antituberculosis	71.4% (5/7)	33.3% (2/6)	54.9% (28/51)	
	Antifungal	85.7% (6/7)	100% (6/6)	100% (51/51)	
	IFN-gama	28.6% (2/7)	16.7% (1/6)	9.8% (5/51)	
Outcome					
	Lost	0% (0/7)	16.7% (1/6)	5.9% (3/51)	
	With HSCT	42.9% (3/7)	20% (1/5)	70.8% (34/48)	
	Without HSCT	57.1% (4/7)	80% (4/5)	29.2% (14/48)	
	Alive without HSCT	100% (4/4)	100% (4/4)	85.7% (12/14)	

*n*, number; *m*, months; *min*, minimum; *max*, maximum; *SMZ*, sulfamethoxazole; *HSCT*, hematopoietic stem cell transplantation; *Frequency of severe infections: serious infection events per patient-year refers to the total number of severe infection events/the duration of the effective observation years

### X91^+^ CGD Gene Mutations and Literature Review

Six missense mutations and one splice site mutation of the *CYBB* gene were detected in the seven patients with X91^+^ CGD. Four X91^+^ CGD–related *CYBB* mutations were reported previously, including c.162G > C, c.170C > A, c.1223G > A, and c.1244C > A, while c.1462–2 A > T and c.1243C > T resulted in amino acid (AA) changes similar to those previously reported at the same location. The c.925G > A mutation has been previously reported to be related to X91^−^ CGD.

**Fig. 4:**
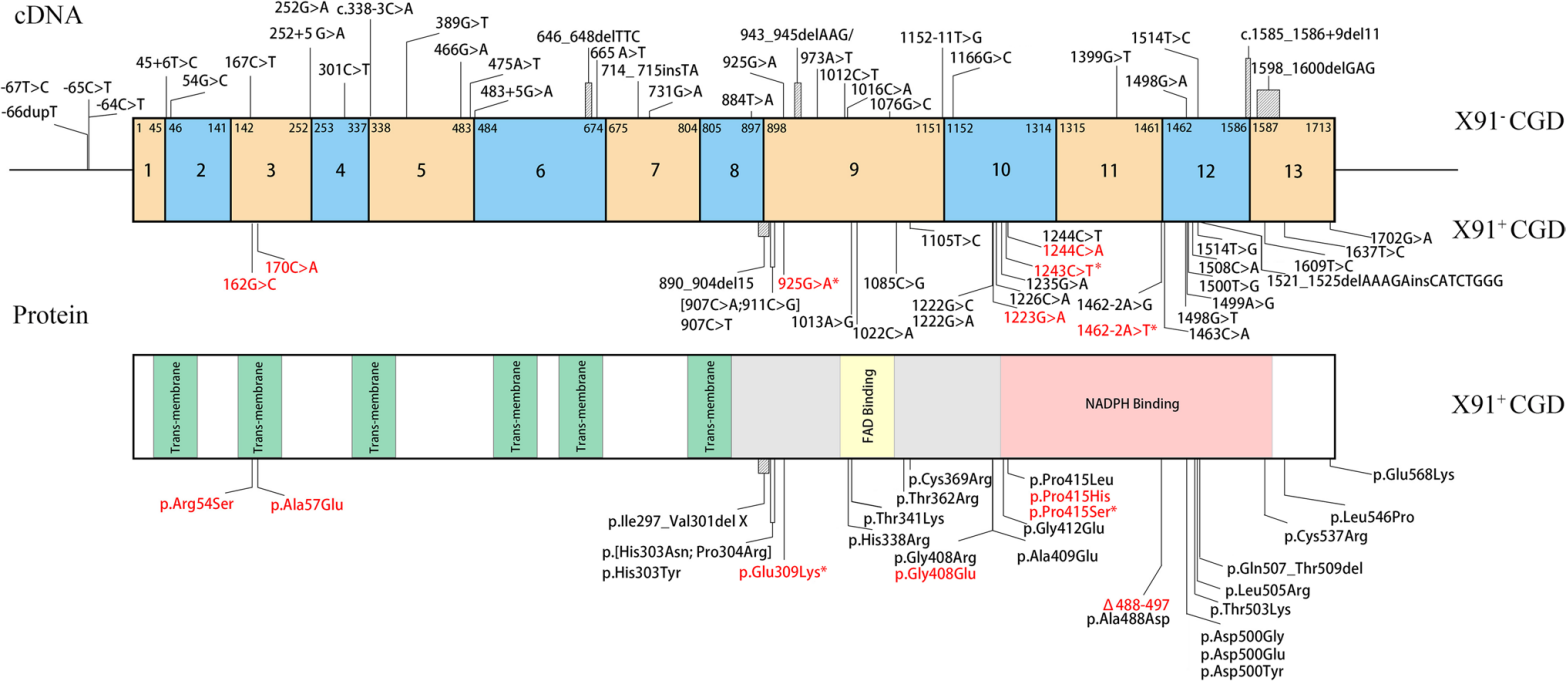
*CYBB* gene mutations in patients with X91^+^ CGD in our cohort and previous reports. The bar above shows the distribution of *CYBB* gene mutations in the cDNA detected in patients with X91^−^ (top panel) and X91^+^ (bottom panel) CGD. The bar below shows the distribution of *CYBB* gene mutations in the protein detected in patients with X91^+^ CGD. The mutation sites identified in our cohort are marked in red. *Novel mutations related to X91^+^ CGD in our study

Then, we searched PubMed and found 35 cases of variant type X91^+^ CGD with available data [1, 2, 4, 8–32], which contained 27 mutations (Table 3). The distribution of loci at the DNA and protein levels is summarized in Fig. 4. Mutations related to X91^+^ CGD, which are mainly located in exons 3, 9, 10, 12, and 13, included 24 missense mutations, one splicing mutation, one deletion mutation, and one deletion/insertion mutation. In the structure of the protein encoded by the *CYBB* gene, two mutations (p. Arg54Ser and p. Ala57Glu) are located in regions encoding the second transmembrane region, most of the other mutations are located in the potential flavin adenine dinucleotide (FAD)– and NADPH-binding domains, and two mutations (p. Cys546Pro and p. Glu568Lys) are located in regions encoding the C-terminal tail, which is essential for NADPH oxidase complex assembly. Therefore, these loci associated with X91^+^ CGD were concentrated in the second transmembrane or intracellular segment.

**Table 3:** CYBB gene mutations reported in patients with variant type X91^+^ CGD

Pt. ID	Nucleotide mutation	Amino acid mutation	MAF in Gnomad	MutationTaster score	SI	NBT	O2- production	GP91phox expression and detection method	cell type	cellular function test	Onset age	Diagnostic age	Age of death	Symptoms	Ref
1	c.162G > C	p.Arg54Ser	0	1	NA	weakly	0	Normal (WB)	Neu	NBT slide test	NA	84 m	NA	Recurrent infection	1, 4, 32*
2	c.170C > A	p.Ala57Glu	0	1	NA	NA	0	Normal (WB)	Neu	chemiluminescence	NA	84 m	NA	Recurrent infection	1, 10, 11*
3	c.890_904del15	p.Ile297_Val301del X	0		NA	NA	0.22 nmol/30 min/2 × 10 ^5^ cells, 20% of normal	Normal (WB)	B cells	cytochrome c reduction	NA	NA	NA	NA	1, 12*
4	c.[907C > A;911C > G]	p.[His303Asn; Pro304Arg]	0	1	NA	0%	0	Normal (spectroscopy and WB)	Neu	NBT slide test, cytochrome c reduction and chemiluminescence	9 m	96 m	NA	Lymphadenitis: *Calmette–Guérin bacillus*, hepatic abscess: *Staphylococcus aureus*	10, 13*, 14*
5	c.907C > T	p.His303Tyr	0	1	NA	NA	0	Normal (WB)	Neu	cytochrome c reduction assay, DHR analysis and bactericidal activity	54y	61y	NA	Skin involvement, liver abscess, pneumonia, osteomyelitis	1, 26*
6	c.1013A > G	p.His338Arg	0	1	NA	45%	Residual	Normal (flow cytometry)	PMN	NBT slide test, cytochrome c reduction assay	3 m	10 m	10 m	Pneumonia: *Burkholderia cepacia*, diarrhea	1, 27*
7	c.1022C > A	p.Thr341Lys	0	1	NA	0	0	Normal (absorption spectroscopy and WB)	Neu	NBT slide test, cytochrome c reduction assay, chemiluminescence	36 m	NA	NA	Pneumonia, lymph nodes infection	1, 15*
8	c.1085C > G	p.Thr362Arg	0	1	NA	NA	NA	Normal (flow cytometry)	Neu	NBT slide test and DHR analysis	NA	NA	NA	*Staphylococcus sciuri, Burkholderia spp.,tuberculosis*	1, 28*
9	c.1105 T > C	p.Cys369Arg	0	1	NA	4%	0	Normal (absorption spectroscopy and WB)	Neu	NBT slide test, cytochrome c reduction assay, chemiluminescence	36 m	NA	NA	Pneumonia: *Aspergillus fumigatus*	1, 4, 15*
10	c.1222G > C	p.Gly408Arg	0	1	NA	4%	NA	Normal (WB)	Neu	NBT slide test	0.2 m	12 m	NA	Pneumonia, hepatic abscess, skin abscess, skin involvement	1, 16*
11	c.1222G > A	p.Gly408Arg	0	1	NA	0	0	Normal (absorption spectroscopy and WB)	Neu	NBT slide test, cytochrome c reduction assay, chemiluminescence	8 m	NA	NA	Pneumonia, lymph nodes infection, liquor: *Mycobacterium bovis*	1,15*
12	c.1222G > A	p.Gly408Arg	0	1	NA	0	0	Normal (WB and differential spectrophotometry)	Neu	NBT slide test and cytochrome c reduction assay	NA	72 m	NA	Pneumonia, sepsis, skin involvement, perianal abscess, colitis, diarrhea, recurrent adenopathies	1, 29*
13	c.1223G > A	p.Gly408Glu	0	1	1	NA	NA	Normal (flow cytometry)	Neu	DHR analysis	NA	NA	NA	NA	2*
14	c.1226C > A	p.Ala409Glu	0	1	NA	NA	0	Normal (flow cytometry)	Neu	DHR analysis	NA	72 m	NA	BCG complications, tuberculosis, subcutaneous abscesses, bronchitis, pneumonia, sinusitis, diarrhea	1, 30*
15	c.1235G > A	p.Gly412Glu	0	1	NA	0	0	Normal (WB)	Neu	NBT slide test and cytochrome c reduction assay	NA	6y	NA	Prolonged fever, pulmonary abcess, Cervical adenopathy, BCGititis, abscess of the anal margin, acute appendicitis, diarrhea	1, 8*
16	c.1244C > A	p.Pro415His	0	1	NA	NA	0	Normal (WB)	Neu	NBT slide test	NA	NA	NA	NA	1, 4, 17, 18*
17	c.1244C > T	p.Pro415Leu	0	1	NA	0	0	Normal (WB), 63% of normal (differential spectrophotometry)	Neu	NBT slide test and cytochrome c reduction assay	NA	18 m	NA	Pulmonary abscess, sepsis, hyperthermia,adenopathy (*S. aureus*), skin inbvolvement	1, 29*
18	c.1462-2A > G	partial del exon 12 p.Ala488_Glu497del	0	1	NA	NA	3.3 (nmol/5 × 10^6^ cells/20 min), 6% of nnormal	Normal (WB and spectrometry)	Neu	cytochrome c reduction assay	69y	69y	NA	DIC, gastational bleeding, renal failure, persistant fever, *pseudomonas cepacia* infection	1, 9*
19	c.1462-2A > G	partial del exon 12 p.Ala488_Glu497del	0	1	NA	NA	NA	NA	Neu	cytochrome c reduction assay	NA	NA	5y	Pneumonia	1, 9*
20	c.1463C > A	p.Ala488Asp	0	1	0.4	0	0	Normal (WB)	Neu	NBT slide test, DHR analysis and bactericidal activity	2y	2y	NA	Persistant lymphadenitis	1, 31*
21	c.1498G > T	p.Asp500Tyr	0	1	NA	0	NA	Normal	Neu	NBT slide test	NA	NA	NA	NA	1, 19*
22	c.1498G > T	p.Asp500Tyr	0	1	NA	0	NA	NA	Neu	NBT slide test	NA	NA	NA	NA	1, 19*
23	c.1498G > T	p.Asp500Tyr	0	1	NA	0	7–9% of normal	Normal (WB)	PMN	NBT slide test, DCFH assay and chemiluminescence	NA	6 m	NA	NA	1, 20*
24	c.1498G > T	p.Asp500Tyr	0	1	6	NA	NA	Normal (flow cytometry)	Neu	DHR analysis	NA	NA	NA	NA	2*
25	c.1499A > G	p.Asp500Gly	0	1	NA	NA	1% of control, 0.13 ± 0.06μmolO2-/min	Normal (WB and spectrometry)	Neu	cytochrome c reduction assay	6 m	36 m	NA	Pneumonia, hepatic abscess, gastational bleeding	1, 21*
26	c.1499A > G	p.Asp500Gly	0	1	8.8	NA	NA	Normal (flow cytometry)	Neu	DHR analysis	NA	NA	NA	NA	2*
27	c.1500 T > G	p.Asp500Glu	0	1	NA	0	0	Normal (WB)	Neu	NBT slide test, DHR analysis and bactericidal activity	NA	2y	NA	Recurrent pyrexia and chronic diarrhea, multiple lymph nodes,peri-anal infections	1, 31*
28	c.1500 T > G	p.Asp500Glu	0	1	NA	0	0	Normal (WB)	Neu	NBT slide test, DHR analysis and bactericidal activity	12 m	12 m	NA	Diarrhea, peri-anal infections,lung abscess	1, 31*
29	c.1508C > A	p.Thr503Lys	0	1	NA	NA	NA	Normal (flow cytometry)	Neu	NBT slide test and DHR analysis	NA	NA	Alive	*S. marcescens* infection	1, 28*
30	c.1514 T > G	p.Leu505Arg	0	1	NA	6%	0	Normal (WB)	Neu	cytochrome c reduction assay	NA	24 m	NA	Sepsis, hepatosplenomegaly	1, 22*
31	c.1521_1525delAAAGA insCATCTGGG	p.(Gln507_Thr509del, insHisIleTrpAla)	0		NA	8%	1%	Normal (WB and flow cytometry)	PMN	NBT reduction assay, cytochrome c reduction assays and chemiluminescence	1 m	36 m	ALive	Pneumonia, perianal abscesses, liver abscesses	23*
32	c.1609 T > C	p.Cys537Arg	0	1	NA	NA	0	Normal (WB)	Neu	DHR analysis, cytochrome c reduction assay and chemiluminescence	NA	NA	NA	NA	1, 17, 24*
33	c.1609 T > C	p.Cys537Arg	0	1	27	NA	NA	Normal (flow cytometry)	Neu	DHR analysis	NA	NA	NA	NA	2*
34	c.1637 T > C	p.Leu546Pro	0	1	NA	NA	2.1–7.2%	Normal (flow cytometry)	Neu	DHR analysis	NA	NA	NA	NA	1, 25*
35	c.1702G > A	p.Glu568Lys	0	1	NA	0	0	Normal (absorption spectroscopy and WB)	Neu	NBT slide test, cytochrome c reduction assay, chemiluminescence	8 m	NA	NA	Sepsis: *Salmonella, Staphylococcus,*	1, 15*

*MAF*, minimum allele frequency; *SI*, stimulation index; *NBT*, nitroblue tetrazolium; *NA*, no available; *WB*, Western blot; *FC*, flow cytometry; *Neu*, neutrophils; *PMN*, polymorphonuclear; *DHR*, Dihydrorhodamine-1,2,3; *DCFH*, Dichlorodihydrofluorescein diacetate; *m*, months; *y*, years

Among 24 patients with X91^+^ CGD for whom definite clinical records of recurrent infection were available, pulmonary infection, including pneumonia and pulmonary abscess, was the most common presentation (14/24). Other clinical manifestations included skin or perianal involvement (9/24), lymphadenitis (7/24), diarrhea (6/24), hepatic abscess (5/24), and sepsis (4/24). One patient experienced disseminated intravascular coagulation (DIC) due to *Pseudomonas cepacia* infection. Although recurrent infection was common, most patients recovered after antibacterial or antifungal treatment. Only two patients died at the ages of 10 months and 5 years due to severe pneumonia.

X91^−^ CGD–related mutations [1, 4, 10, 17, 18, 20, 22, 25, 33–57] included splicing, deletion, and missense mutations. X91^−^ CGD involved mutations across all domains and was not limited to the specific segment as observed in X91^+^ CGD.

## Discussion

CGD is a rare primary immunodeficiency resulting from the inability of neutrophils to generate respiratory bursts and produce O_2_^−^. *CYBB* mutations encoding gp91phox result in X-linked CGD. Typical X-linked CGD accounts for more than 90% of X-linked CGD cases [10], with a pattern of early-onset disease characterized by lack of gp91phox expression and low neutrophil SI. Therefore, the combination of low SI and lack of gp91phox expression is often used in the rapid clinical diagnosis of X-linked CGD. Approximately 80% (51/64) of patients in our cohort had X91^0^ CGD, while other patients had specific GP91 protein signatures. The proportion of X91^+^ CGD was higher than expected.

Gp91phox is composed of 570 amino acids. The N-terminus contains six transmembrane regions, in which two hemes are coordinated by four histidines. The C-terminus of gp91phox is a cytosolic tail containing NADPH- and FAD-binding sites. The C-terminal cytosolic tail of gp91phox is responsible for transferring electrons from intracellular NADPH to FAD, and this reaction activity is called iodonitrotetrazolium (INT) diaphorase activity. Two hemes are aligned in series and are responsible for transferring electrons from reduced FAD (FADH2) to extracellular or intravesicular O_2_, thus forming O_2_^−^. Resting-state gp91phox binds to p22phox and localizes to the cellular membrane. After receiving activation signals, p47phox, p40phox, and p67phox translocate to the gp91phox-p22phox heterodimer to form the NADPH oxidase complex [58]. Western blotting was used to analyze gp91phox in the past, while flow cytometry–based extracellular staining with monoclonal antibody (mAb) 7D5 is currently used to analyze gp91phox levels [1, 2].

The review showed that X91^+^ CGD–associated mutations were often located in the second transmembrane or intracellular regions, including potential FAD/NADPH-binding domains, which may be hot spots. Different mutation sites lead to different changes in protein function. Mutations p. Arg54Ser and p. Ala57Glu are located at the second transmembrane region of gp91phox. Positively charged Arg54 is hydrogen bonded to the outer heme. Mutated gp91phox with uncharged Ser54 has a redox potential of − 300 mV, thus blocking electrons exiting from heme to form O_2_^−^ [59]. In vitro experiments confirmed normal FADH2 levels [60] but no O_2_^−^ production by the Arg54Ser mutation [17]. A similar mechanism is observed for p. Ala57Glu. Ala57 is an uncharged amino acid; however, glutamic acid is negatively charged and much larger than Ala, thus inhibiting electron exit from the transmembrane passage [11]. The mutation p. Glu309Lys is located in the region between the membrane and dehydrogenase domain and is involved in p65-p22phox binding [61]. P415 is located at the intracellular NADPH-binding region of gp91phox. The mutation p. Pro415His disrupts the consensus sequence for an NADPH-binding site (GXGXGXXPF) and causes NADPH binding defects [62, 63]. Therefore, p. Pro415His-mutated gp91phox does not produce O_2_^−^, although gp91phox expression levels, FAD incorporation, and NADPH oxidase assembly are normal. Presumably, p. Pro415Ser results in similar changes to p. Pro415His. Studies suggested that the p. Gly408Glu mutation strongly inhibited the translocation of p47phox and p67phox to the phagosomal membranes of the mutants [62]. Defective FAD incorporation results in a deficiency in NADPH oxidase activity. This mutation presumably causes defective complex assembly, directly interferes with FAD/NADPH binding, and disrupts electron transfer [62, 63]. According to the analyses of the previously reported mutations c.1462-2A > G and c.1462-2A > C, we hypothesize that the c.1462-2A > T mutation removes the 3′ splice acceptor site of intron 11 and results in a partial p. Ala488_Glu497del mutation in the gp91phox protein [1, 9]. In wild-type gp91phox, residues 484 to 503 form an α-helical loop lying over the NADPH-binding cleft [31]. For patients carrying the c.1462–2 mutation, loss of this structure subsequently disturbs NADPH binding and electron transfer to FAD. Therefore, the normally expressed gp91 protein is nonfunctional or partially functional and results in defective neutrophils in these patients.

Notably, a high neutrophil SI detected using the DHR123 test does not indicate mild symptoms or a good prognosis for patients with variant type X-linked CGD. Although DHR has the advantages of requiring a small blood volume, short time, and convenience compared with direct superoxide detection, it has limitations in terms of molecular mechanism [64]. NADPH oxidase transfers electrons to molecular oxygen to generate O_2_^−^, which is dismutated to hydrogen peroxide (H_2_O_2_). Although to a lesser extent and lower priority, electrons can leak from FADH2 in the NADPH oxidase enzyme, directly producing H_2_O_2_ without the intermediate O_2_^−^. For neutrophils with normal FADH2 but no O_2_^−^, such as p. Arg54Ser, neutrophil respiratory burst tests indicate the H_2_O_2_ produced by FADH2, and thus the SI does not fully reflect O_2_^−^ levels which represent the function of NADPH oxidase [32]. The partial production of H_2_O_2_ generation is not sufficient to protect patients with X91^+^ CGD from severe infections, under conditions with a high bacterial load [26]. In our study, all patients with CGD had a lower SI than healthy controls. Patients with X91^+^ variant–type CGD could have a higher SI than patients with X91^−^ or X91^0^ CGD, but the three groups showed no significant differences in onset age, diagnosis age, or severe infection frequency. The SI was not statistically correlated with the age of onset or the frequency of severe infections. In the cohort analyzed by Wu et al., a patient with an SI of 40.15 died of severe pneumonia within 1 year of age, while a patient with an SI of 1.99 was still alive at 10 years of age [46]. Therefore, the SI may have more value in the diagnosis of CGD than in the prediction of disease severity. The diagnosis of CGD should be considered when SI is below the normal range. For patients with X91^+^ CGD and a slightly lower SI, physicians should evaluate their manifestation and make the diagnosis carefully to avoid delay. Bacterial killing tests and superoxide quantification tests should be performed if necessary.

Interestingly, the same mutations in different patients sometimes result in different phenotypes. For example, the mildest case of *CYBB* c.1462–2 A > G mutation remained healthy until the age of 69 years, while one of his grandsons died at the age of 5 years [9]. In our cohort, male patients in the family of P7 also presented the disease with varying severity. Two uncles and his oldest brother died of fever and lung infection within 5 years after birth, while P7 and his second brother survived to the ages of 8 and 16 years, respectively. Similar conditions were reported in patients carrying other mutations [10, 23, 55], indicating that other factors are involved in the immune defense of CGD patients [1, 9, 26, 65]. Clinical symptoms, SI, protein expression, and gene analysis should be considered for a comprehensive evaluation of patients with variant type X-linked CGD, especially X91^+^ CGD.

## Supplementary Information

Below is the link to the electronic supplementary material.

Supplementary file1 (DOCX 24 KB)

## Data Availability

The datasets generated and analyzed during the current study are available from the corresponding author upon reasonable request. The study was approved by the ethics committee of Children’s Hospital of Fudan University.
