# Supplementary material for: Variant Type X91+ Chronic Granulomatous Disease: Clinical and Molecular Characterization in a Chinese Cohort
Source: J Clin Immunol. 2022 Jul 7;42(7):1564–79. doi: 10.1007/s10875-022-01324-3 (PMC9674757; doi:10.1007/s10875-022-01324-3)
Supplement: Supplementary file 1 — Supplementary file1 (DOCX 24 KB) [file 10875_2022_1324_MOESM1_ESM.docx]

**Supplemental 1: Patient History**

The individual characteristics of seven patients with X91^+^ CGD are described in detail below, including age, sex, clinical manifestation, molecular diagnosis, treatments, and family history.

Patient 1:

P1 is a 25-month-old Chinese boy. His mother had an elder brother who died at ten years of age for unknown reasons. The patient received the BCG vaccine at birth and developed left axillary lymph node enlargement after three months, and his skin lesion at the BCG vaccine injection site did not heal until he was six months old. At eight months of age, this patient was hospitalized due to recurrent fever, cough, diarrhea, and finger and foot swelling. The routine blood test showed elevated white blood cell (WBC) counts of 18.32×10^9^/L and C-reactive protein (CRP) levels of 31 mg/L. Computed tomography (CT) indicated multifocal pneumonia, and *Clostridium difficile* infection was identified in the stool. The patient was treated with antibiotics such as ceftriaxone, piperacillin-tazobactam, metronidazole and fluconazole. The soft tissue swelling was improved with additional vancomycin treatment. However, he experienced a recurrent fever after 1 month of treatment. The lung CT manifestation showed multiple masses, and the galactomannan (GM) test of bronchoalveolar lavage fluid (BALF) was positive. After treatment with caspofungin and voriconazole successively, the child's temperature gradually stabilized. A few days later, the child developed a fever with obvious foot swelling. *Burkholderia gladioli* (reads, 69) and *Mycobacterium neoaurum* (reads, 78) were identified in the pus from the right foot and blood, respectively, using metagenomic sequencing (mNGS). Magnetic resonance imaging (MRI) and bone scanning confirmed multifocal bone destruction and osteomyelitis involving his left finger, right lower leg, and foot. Liver enzyme levels were transiently elevated. The patient was in a stable condition after treatment with antibiotics, including meropenem, vancomycin, voriconazole, sulfamethoxazole (SMZ), etc., and intravenous immunoglobin. A subsequent immune function analysis showed that his SI was 67.3 and that he had a normal gp91phox expression level. At 11 months of age, gene testing showed a missense mutation in the *CYBB* gene: c.162 G>C, p.Arg54Ser. His mother was a carrier, and her SI was 352.2. The child was treated with hematopoietic stem cell transplantation (HSCT) at 13 months old (chimerism of 99.69% at 14 days). The child was generally in good health during the one-year follow-up after HSCT.

Patient 2:

P2 was diagnosed with CGD at seven years old. He had no significant family history. He experienced a recurrent aphthous ulcer every one to two months since he was 1 year old without fever or diarrhea. At the age of three years, the patient developed one nodule on the left forearm that gradually ruptured and was purulent. The pus cultures were negative, and the tissue pathology suggested infectious granuloma. The ulcer healed after clarithromycin and rifampicin treatment for 2.5 months. One year later, he developed a mass with tenderness in his left cervical region, and the pus culture was negative. The patient was diagnosed with cervical lymphadenitis with abscess formation. Surgical and amoxicillin therapy were applied with a satisfying outcome. He experienced recurrent fever and cough at seven years old. A routine blood examination indicated elevated WBC counts of 13.6×10^9^/L and CRP levels of 68.1 mg/L. Chest CT showed left upper pneumonia and atelectasis, and no pathogens were detected in BALF using mNGS. He initially received azithromycin, cefoperazone-sulbactam, rifampin, clarithromycin, and SMZ, and then voriconazole and interferon-gamma (IFN-γ) were added after an immunologic evaluation. Finally, the child's temperature gradually decreased to normal. A routine analysis of immune function, including numbers of lymphocyte subsets and immunoglobulin levels, was normal. His SI was 35.4, while his gp91phox expression was normal. The SIs of his father and mother were 158.0 and 102.0, respectively. The genetic analysis reviewed a*de novo* variant in the *CYBB* gene: 1462-2 A>T. He received regular IFN-γ injections and treatment with anti-infective agents such as SMZ and itraconazole. The patient is now 11 years old and has no apparent infection.

Patient 3:

P3 is now a 46-month-old boy without a clear family history. His mother had no pregnancy or abortion before his birth. He was hospitalized for one week after birth because of meconium aspiration pneumonia and was not vaccinated. He was admitted to the hospital again due to a rough cough and eczema at 20 days of age. Continuously elevated WBC counts (>20×10^9^/L) were detected. The lung CT showed masses in the lower left lung with negative sputum culture and T-spot test. Ultrasound indicated enlarged superficial lymph nodes. His alanine transaminase (ALT) level was slightly elevated. He experienced recurrent hospitalizations and was treated with various antibiotics, including cefoperazone/sulbactam, moxalactam, imipenem, vancomycin, fluconazole, dexamethasone, and IVIG infusion. Finally, the cough symptoms improved, and the lung inflammation was apparently absorbed. Testing the respiratory burst of neutrophils showed that the SI was 21.2, and the expression level of gp91phox was normal. At four months of age, genetic testing indicated a maternal mutation in *CYBB*: c.170C>A, p.Ala57Glu. After the diagnosis was confirmed, itraconazole and SMZ were added orally to prevent infection. The child was treated with HSCT at one year old (chimerism of 100% at ten days).

Patient 4:

P4 was 22 months old when he visited our hospital, and he is now 31 months old. He was vaccinated with BCG immediately after birth. His parents and two sisters were healthy. Beginning at the age of 4 months, the patient presented intermittent fever, left axillary lymph node enlargement, and perianal abscess. Lung CT showed bilateral lung infection, and the PPD test was negative. Each fever lasted 1-5 days, and the body temperature decreased to normal after five days of cephalosporin treatment. Reexamination of the PPD test at 15 months revealed a strong positive result. He received triple anti-tuberculosis medications, including isoniazid, rifampicin, and ethambutol. Two months later, the abdominal CT indicated spleen enlargement and peritoneal effusion, and then linezolid was added. After four months of anti-tuberculosis treatment, his parents stopped the medications. Then, the child developed recurrent fever once a month, perianal abscess, and multiple lymph node enlargements. However, acid-fast bacilli were not detected in gastric fluid, BALF, or punctured lymph nodes. *Ureaplasma urealyticum* was also detected in sputum using PCR, and *Corynebacterium striatum* (reads, 200) was detected in BALF using mNGS. Colonoscopy showed colitis and proctitis. After treatment with cefoperazone/sulbactam, azithromycin, isoniazid, itraconazole, SMZ, and topical drugs, the child's body temperature gradually stabilized. The patient’s lymphocyte subsets and immunoglobulin levels were normal. The respiratory burst of neutrophils measured using the flow cytometry-based DHR test showed an SI of 1.9, and the gp91phox expression level was normal. A maternal mutation in *CYBB*: c.1223G>A, p. G408E was detected using WES. Currently, the patient is taking oral antibiotics for subsequent transplantation treatment.

Patient 5:

P5 is now a boy aged approximately 11 years who received the BCG vaccine at birth, and five months later, he presented with fever and local suppuration at the vaccination site. Subsequently, the child developed lymphadenopathy of the left clavicle and scapula. The lymph node pathological examination in the local hospital suggested tuberculosis, and thus isoniazid and rifampicin were added to the treatment for one year. The patient had bilateral aural fistula oozing pus and bronchitis or upper respiratory tract infection once or twice a year. When he was seven years old, he developed recurrent fever, mainly low fever with a peak temperature of 37-38 °C, accompanied by painful cervical lymph node enlargement. Puncture pathology indicated lymph node tuberculosis, although no definite *Mycobacterium* was detected by mNGS. He was administered isoniazid, rifampicin, pyrazinamide, and ethambutol for anti-tuberculosis treatment and underwent lymph node dissection. After admission to our hospital, the PPD test indicated an induration of 1.0*1.1 cm. His chest CT revealed multiple noticeable calcifications of cervical and axillary lymph nodes. The respiratory burst of neutrophils measured using the flow cytometry-based DHR test showed an SI of 40.7. WES indicated a *de novo* mutation in the *CYBB* gene: c.1243C>T, p. P415S.

Patient 6:

P6 is a 46-month-old boy. The patient received the BCG vaccine at birth. On the 15th day after birth, the child presented recurrent fever. The routine blood examination indicated a significant increase in WBC counts and CRP levels, and chest CT revealed multiple sites of inflammation with abscess formation in both lungs. Anti-infection medications, including ceftriaxone, meropenem, fluconazole, and IVIG infusion, were administered. A genetic test was performed, indicating a mutation in the *CYBB* gene: c.1244C>A, p.P415H. At two months, the child developed a recurrent perianal abscess, increased stool frequency, and then fever again, which improved after ceftriaxone and vancomycin anti-infection treatment. At four months, the child experienced a fever again, and SMZ was added to prevent *Pneumocystis carinii* infection. At seven months, the child was hospitalized in our hospital with recurrent fever accompanied by inguinal abscess and cough. The DHR test showed an SI of 17.1. The lung CT suggested diffuse changes that were accompanied by a positive PPD test (1.5 cm*1.5 cm), and the abscess culture in the groin area suggested the presence of *Klebsiella pneumonia*. The patient was treated with cefoperazone/sulbactam, linezolid, SMZ, isoniazid, and rifampicin, and his condition improved gradually. At 15 months of age, he was treated with HSCT, and the chimerism rate was 99.92% at Day 14 after transplantation.

Patient 7:

P7 had a positive family history. His two uncles and eldest brothers died of fever and lung infections at three, five, and five years old, respectively. After BCG vaccination, the second brother exhibited axillary lymph node enlargement, followed by repeated pulmonary tuberculosis and lymph node tuberculosis. P7 received the BCG vaccine after birth and had no obvious adverse reactions. At age two, he was diagnosed with bronchitis due to recurrent fever and hospitalized for a week. When he was four years old, P7 developed continuous fever for more than one month, accompanied by rash and enlarged lymph nodes in the neck. Lung CT suggested multiple miliary nodules and enlarged lymph nodes in the neck. Acute miliary tuberculosis was diagnosed locally. The patient was administered anti-infection treatment with isoniazid, rifampicin, pyrazinamide, ethambutol, and cefoxitin, and his condition improved gradually. Isoniazid and rifampicin were continually used after discharge. Later, oral ulcers and pharyngeal pain occurred once a month, and oral cephalosporin antibiotics were administered. When he was five years old, he received medical treatment in Shanghai, and the genetic test revealed a maternal mutation in the *CYBB* gene: c.925G>A, p. E309K. The child received regular anti-tuberculosis treatment for more than one year, and repeated fever occurred two weeks after self-withdrawal, with left submaxillary lymph node enlargement of 2.0*2.0 cm, and the PPD test was positive. He was treated again with isoniazid, rifampicin, and ethambutol for anti-tuberculosis treatment, itraconazole to prevent fungal infection, cefoperazone sodium and sulbactam sodium for antibacterial treatment, and IFN-γ injections. The child is now nine years old, and the infection was under control during a recent telephone follow-up.
